# Genetic study of congenital bile-duct dilatation identifies de novo and inherited variants in functionally related genes

**DOI:** 10.1186/s12920-016-0236-z

**Published:** 2016-12-12

**Authors:** John K. L. Wong, Desmond Campbell, Ngoc Diem Ngo, Fanny Yeung, Guo Cheng, Clara S. M. Tang, Patrick H. Y. Chung, Ngoc Son Tran, Man-ting So, Stacey S. Cherny, Pak C. Sham, Paul K. Tam, Maria-Mercè Garcia-Barcelo

**Affiliations:** 1 Department of Psychiatry, Li Ka Shing Faculty of Medicine, The University of Hong Kong, 1F Room 5D HKJCBIR, 5 Sassoon Road, Hong Kong, SAR China; 2 National Hospital of Pediatrics, Hanoi, Vietnam; 3 Department of Surgery, Li Ka Shing Faculty of Medicine, The University of Hong Kong, Hong Kong, SAR China; 4 Center for Genomic Sciences, Li Ka Shing Faculty of Medicine, The University of Hong Kong, Hong Kong, SAR China; 5 Centre for Reproduction, Development, and Growth, Li Ka Shing Faculty of Medicine, The University of Hong Kong, Hong Kong, SAR China

**Keywords:** Choledochal cyst, Exome, De novo, Rare variants association

## Abstract

**Background:**

Congenital dilatation of the bile-duct (CDD) is a rare, mostly sporadic, disorder that results in bile retention with severe associated complications. CDD affects mainly Asians. To our knowledge, no genetic study has ever been conducted.

**Methods:**

We aim to identify genetic risk factors by a “trio-based” exome-sequencing approach, whereby 31 CDD probands and their unaffected parents were exome-sequenced. Seven-hundred controls from the local population were used to detect gene-sets significantly enriched with rare variants in CDD patients.

**Results:**

**Twenty-one predicted** damaging de novo variants (DNVs; 4 protein truncating and 17 missense) were identified in several evolutionarily constrained genes (*p* < 0.01). Six genes carrying DNVs were associated with human developmental disorders involving epithelial, connective or bone morphologies (*PXDN, RTEL1, ANKRD11, MAP2K1, CYLD, ACAN)* and four linked with cholangio- and hepatocellular carcinomas *(PIK3CA, TLN1 CYLD, MAP2K1)*. Importantly, CDD patients have an excess of DNVs in cancer-related genes (*p* < 0.025). Thirteen genes were recurrently mutated at different sites, forming compound heterozygotes or functionally related complexes within patients.

**Conclusions:**

Our data supports a strong genetic basis for CDD and show that CDD is not only genetically heterogeneous but also non-monogenic, requiring mutations in more than one genes for the disease to develop. The data is consistent with the rarity and sporadic presentation of CDD.

**Electronic supplementary material:**

The online version of this article (doi:10.1186/s12920-016-0236-z) contains supplementary material, which is available to authorized users.

## Background

Congenital dilatation of the bile ducts (choledochal cyst/CDD) result in bile retention, with cholangitis, pancreatitis and malignancies as associated complications. The main symptoms are cholestatic jaundice, abdominal pain and liver enlargement. If the obstruction is not relieved the liver can be permanently damaged [1, 2]. Surgery is the only available treatment. CDD is a rare, mostly sporadic, disorder whose incidence varies widely among populations (from 1 in 1,000 in Asians to 1 in 150,000 in Caucasians). CDD is anatomically classified in 5 major types (I-V) [3]. Types I and IV denote dilatation of the common biliary duct (CBD) without (type I) or with (type IV) dilated intrahepatic ducts and are the commonest, representing 50%–80% and 15%–35% of the CDD paediatric population, respectively. Type II CCDs appear as a diverticulum protruding from the wall of the CBD and Type III as a dilatation of the duodenal portion of the CBD. Type V or Caroli disease, refers to a congenital polycystic dilatation only affecting intra hepatic biliary ducts. Type V is considered as a distinct entity as, unlike the other types, is usually associated with both cystic renal disease and liver fibrosis (Caroli syndrome).

There are two main theories on the aetiology of Types I and IV CDD, the “obstructing segment” hypothesis and the “pancreatic reflux” hypothesis. The former suggests that a congenital stenosis of the distal bile duct increases the intralumenal pressure which, in turn, dilates the proximal part of the choledoch. The latter proposes that CDD is caused by reflux of pancreatic enzymes into the CBD as a result of an anomaly of the pancreaticobiliary junction. Mixing and activation of the pancreatic and biliary secretions deteriorate the CBD wall and lead to dilatation [4, 5].

Both theories imply the presence of congenital structural anomalies that are likely to reflect a failure in the hepatobiliary-pancreatic development during embryonic stages. Although familial cases of CDD have been reported [6], the overall evidence for a classic genetic inheritance of the phenotype is scant. Nonetheless the, i) the female/male bias observed for Types I and IV CDD; ii) the marked difference in incidence among populations; iii) the rarity of the disorder together; iv) the presence of congenital structural anomalies; and the fact that CDD types I or V can be associated with familial adenomatous polyposis [7] and autosomal recessive or dominant polycystic kidney disease (PKD) respectively [8], are strong indicators of a genetic contribution to CDD. Yet the scarcity of pedigrees segregating the disorder and the rarity of the disease make identification of genetic risk loci by traditional study designs difficult. Conceivably, DNA alterations in genes governing the embryonic development of the hepatobiliary-pancreatic system could underlie CDD.

Given all of the above, we set out to investigate the genetic basis of CDD under the hypothesis that rare de novo or recessive inherited damaging genetic variants could trigger the disorder and account the sporadic presentation of CDD, the scarcity of familial cases and the variability in its incidence across populations. For the identification of loci underlying “sporadic” (no family history) genetic disorders, the best strategy is the trio-based approach whereby the exome of unaffected parents and affected probans is scrutinized [9]. Known DNA-variants are filtered out leaving researchers with DNA-variants/mutations unique to the trio (both de novo and inherited). Parents/child filtering is used to identify de novo mutations.

## Methods

### Study design

We adopted the “trio-based” whole exome-sequencing approach by which the exomes of the patient and his/her unaffected parents are analysed.

Common DNA-variants were filtered out leaving only variants whose minor allele frequency (MAF) in the general population is < =1%. This is consistent with recessive transmission of the disorder and in line with its incidence in Asia (Additional file 1). Comparison of parental and offspring sequence was used to identify de novo variants.

The study was approved by the institutional review board of The University of Hong Kong together with the Hospital Authority (IRB: UW 07–321). Blood samples were drawn from all participants after obtaining informed consent (parental consent in new-borns and children below age 7) and experiments were carried out in accordance with the approved guidelines.

### Patients

A total of 33 CDD ethnic Chinese patients (1 male and 32 females) with dilatation of the common biliary duct and their parents were included in the study. Patients from Hong Kong (*N* = 25 trios; 1 male and 24 females) were recruited through our specialist clinic to which all patients in the territory are referred. Eight additional ethnic Chinese trios (8 females) were recruited through the National Hospital of Pediatrics in Hanoi, Vietnam.

Patients were diagnosed antenatally, by ultrasound, or postnatally by magnetic resonance cholangiopancreatography (MRCP). Clinical characteristics of the patients are detailed in Additional file 1: Table S1.

### Controls

For association tests and quality assessment, data from 700 local Chinese participating in a degenerative disc disease (DDD) in-house exome sequencing project were added to the calling set (Additional file 1).

### Procedures

#### Whole Exome Sequencing (WES) and bioinformatics analyses

Additional file 1.

#### Testing enrichment of genes mapped on significantly mutated regions (SMRs) in cancers

A recent study has summarized significantly mutated regions (SMRs) in 21 types of cancer [10]. These mutated regions overlap among different type of cancers and provide insights into the molecular mechanisms underlying tumorigenesis. To identify susceptibility genes shared by cancer and CDD, we tested our samples for enrichment of de novo mutated SMR genes. A total of 610 SMR genes listed on the paper were retrieved and the test was performed by hypergeometric test.

#### Tests on inherited variants: homozygosis, compound and di-genic heterozygosity

We hypothesised monogenic and digenic recessive inheritance models for the disease. We considered the following i) homozygous variations; ii) compound heterozygous (CH), and iii) variants in two different but interacting genes (PPIs). For each scenario, we only considered variants inherited from different parents, that is, we required both maternal and paternal rare alleles, which would fit the mostly sporadic presentation of CDD. Since rare variants are more likely to cause disease, allele frequency was restricted to < =1% MAF giving an incidence for any such combination in an individual of < =0.01%.

However gene length would affect how many of these rare mutations might be seen in the parents in a given gene, as would population specific linkage disequilibrium. To account for these two issues and to calculate the genetic burden of each CH or PPI event in patients we performed case–control association tests whereby the co-occurrence of two variants per individual were compared.

For CH and homozygous events, we tested the burden of genes containing > =2 damaging variants on each single individual using Fisher exact test. CH and homozygous events with *p*-values >0.01 were removed.

For PPI interacting pairs with a rare variant each, we filtered out those PPI pairs with *p*-values >0.01 in their respective set-based analysis (described above). To further narrow down our results, only recurrent PPI events were included in these tests.

#### Gene based and set based association tests

Enrichment tests on rare damaging variants were performed at gene level. A Kernel style association test, the **S**equence **K**ernel **A**ssociation **T**est (SKAT) [11] was chosen for the purpose. The test was carried out by the program RVTEST (rare variant test software for next generation sequencing data), which implements the SKAT test and accepts gene-based as well as set based tests (PPI -protein-protein interactions- and pathways) as options. Only SNVs with MAF < = 1% in the 1000 Genome project, ESP6500 and dbSNP137 databases were included. The same criterion was applied to SNVs of our in-house exome dataset (exomes of 700 Chinese individuals).

To check the association results for batch bias, controls (*N* = 700) were compared with pseudo-controls (*N* = 52) derived from other trio-based in-house sequencing study. These pseudocontrols had been sequenced in the same batch and processed using the same analysis pipeline as our cases. Since the pseudocontrols also represent the normal population, SNVs overrepresented in any of the groups being compared (controls vs. pseudocontrol) were indicative of batch bias and were therefore removed from the call set.

For gene-based association tests, genes carrying 3 or fewer variants were excluded from the analysis. The removal of genes with insufficient markers can increase power by reducing the number of multiple testing corrections.

For set-based association tests, we considered groups of genes with similar biological functions as the unit of testing. In these tests, we used multiple sources of biological knowledge including protein-protein interactions (PPI) and curated pathways. Gene-sets linked by PPI were retrieved from STRING v9.5 database [12]. Only PPI pairs with medium experimental evidence or above (threshold of 0.4 according to STRINGDB website) were used. Genes carrying filtered SNVs were used to produce these PPI pairs, we included only PPI-sets with 5 or more markers in the tested sets.

Pathway sets for testing were retrieved from MSigDB, KEGG, Biocarta and REACTOME. Similar to the PPI based analyses, we included only gene-sets with 5 or more markers in each test. In total there were ~9000 PPI pairs and 1056 pathways being tested by SKAT, resulting *p*-values were corrected by the number of PPI/Pathway sets tested.

## Results

### Sample processing

We conducted a whole exome sequencing (WES) analysis of 33 CDD trios to identify causal genetic variations within coding regions. All patients had a normal karyotype.

There is a remarkable difference of sex ratio in our sample set (female:male = 32:1). This ratio does not reflect that of our population [2, 13] but the selection process of the patients included in the study which was dependent on the availability of parental DNA (trios) and willingness to participate.

Two trios were excluded from the analysis due to DNA contamination and non-maternity relationship, respectively. Thus, 31 trios were analysed.

### Analysis

As pathological mechanisms we considered the effect of damaging de novo germ-line mutations and rare recessive inherited variants in i) homozygosis; ii) compound heterozygosis (CH; different mutations in the same gene, one paternally and one maternally inherited and iii) a “di-genic model” assuming that variants in different genes of related pathways coexist in the patient through maternal and paternal inheritance. The combined effect of several variants (either de novo + inherited or inherited) was also considered.

We also performed case–control association tests to capture the possible enrichment of rare variants in a given gene in the patients. Gene-based and, the more powerful, gene-set/pathway-based tests were carried out to identify genes with damaging alleles that, alone or together with their interacting partners, are associated with CDD.

In total, predicted damaging variants were found in 34 genes, and these were distributed among 23 trios. In the remaining 8 trios no damaging variants were identified (Additional file 1: Table S2).

#### De novo variants

We have identified 27 non-synonymous de novo variants among 31 trios through our pipeline, 24 of them were validated by Sanger sequencing, achieving an 89% validation rate. Among the validated de novo variants, 21 were predicted damaging (4 protein truncating and 17 missense mutations) and mapped to the coding sequences (CDS) of 21 genes of 19 CDD patients (Table 1). The de novo damaging CDS variant rate is 0.6 per patient and that of the overall de novo CDS mutations (damaging and not damaging) is 1.1 per patient. The non-synonymous to synonymous ratio (NS:S) of de novo variants is 3:1 which is comparable to a reported random model (NS:S = 2.85:1) [14] and slightly higher than the ratio reported by 1000 Genomes Project (NS:S = 1.14:1) [15].

**Table 1:** List of de novo damaging variants

Family	Genes	Variant type	Protein change	DNA Change	Associated human disease	Mouse phenotype^a^
CC14	*PQLC2*	M	p.V2F	c.4G > T	NA	NA
CC205	*PTGER3*	M	p.A158V	c.473C > T	NA	NA
CC226	*ACAN*	M	p.C2282R	c.6844 T > C	Spondyloepimetaphyseal dysplasia (AR), Osteochondritis dissecans	Abnormal liver morphology, enlarged liver
CC234	*KRT80*	M	p.S249C	c.745A > T	NA	NA
CC3	*TENM4*	M	p.R2238W	c.6712C > T	NA	NA
CC35	*TMEFF1*	M	p.H231R	c.692A > G	NA	NA
	*PXDN*	F	p.P225fs	c.673delC	Sclerocornea (AD)	NA
CC4	*MAP2K1*	M	p.I103S	c.308 T > G	Noonan's syndrome (AD, AR)	NA
CC55	*ANKRD11*	M	p.K1464fs	c.4388del-GAGA	KBG syndrome (AD)	NA
	*TLN1*	M	p.R2398W	c.7192C > T	NA	NA
CC7	*PPP2R2B*	F	p.G386fs	c.1128del-G	Spinocerebellar ataxia (AD)	NA
CC81	*C6*	M	p.W571C	c.1713G > T	Complement component 6 deficiency	NA
	*HEATR6*	M	p.T948I	c.2843C > T	NA	NA
	*PIK3CA*	M	p.I191M	c.573A > G	Keratosis seborrheic (AD), cancer	Increased pancreatic beta cell number
VC10	*PPP1R15B*	M	p.P139H	c.416C > A	NA	Abnormal liver morphology
	*RTEL1*	S	p.G973^b^	c.2917G > T	Dyskeratosis congenital (AR/AD), Pulmonary fibrosis (AR)	NA
	*TXLNB*	M	p.R211Q	c.632G > A	NA	NA
VC61	*CYLD*	M	p.W487L	c.1460G > T	Familial multiple trichoepitheliomata; Spiegler-Brooke syndrome (AR)	Abnormal intestinal epithelium morphology
	*KCNH3*	M	p.V195A	c.584T > C	NA	NA
	*SDC3*	M	p.R302W	c.904C > T	NA	NA
VC63	*ZNF330*	M	p.F54L	c.160T > C	NA	NA

*AR* autosomal recessive, *AD* autosomal dominant, *M* missense, *S* stopgain, *F* frameshift, *NA* not described

^a^hepatobiliary/pancreas

^b^HGVS standard for stop-codons

**Fig. 1:**
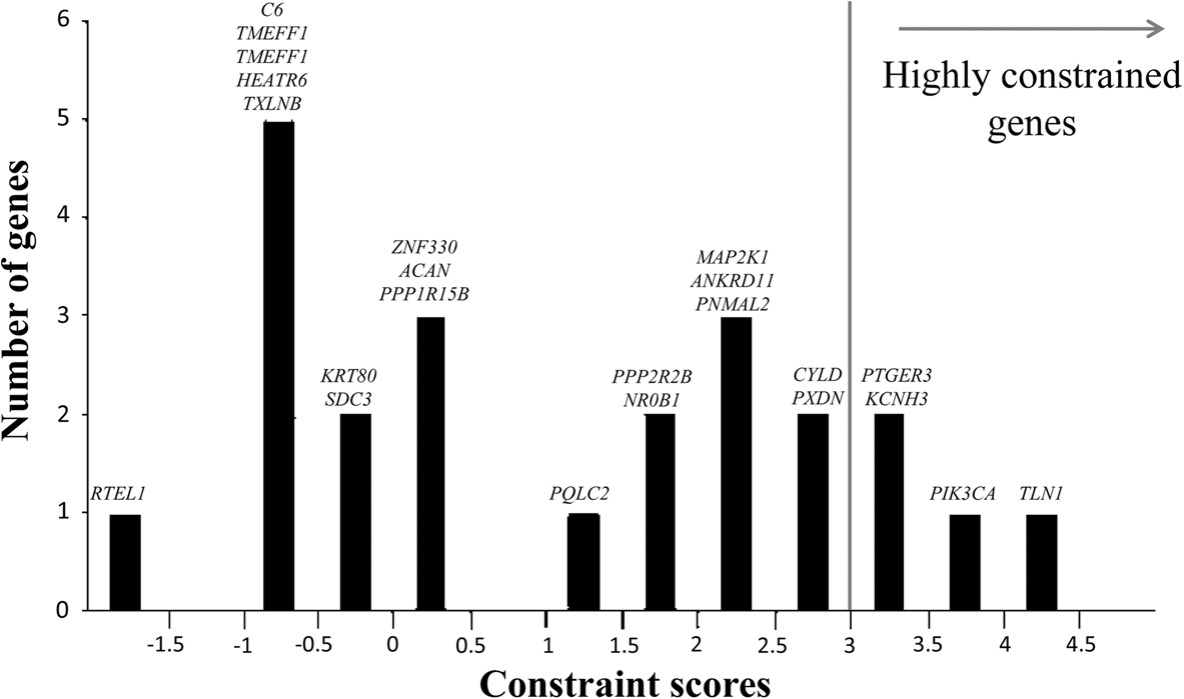
Distribution of constraint scores of all genes with de novo non-synonymous variants. Y axis: number of genes whose constrain scores fall into the range specified in the X axis. X axis: constrain scores as defined by Samocha et al. [16]. (The genes with constraint score > 3.09 are highly constrained, corresponding to *P* < 0.001 and represented roughly 5% of all genes.)

Non-synonymous de novo variants were annotated (Table 1) and their Constraint Scores [16] (Additional file 1: Table S2) calculated (Fig. 1). The plot shows that the gene constraint scores deviate from the empirical normal distribution to a bimodal distribution with a set of 4 genes being evolutionary constrained (constrain score cut-off = 3.09, corresponding to *P* < 0.001). The average constraint score of 21 damaging de novo variants is significantly higher than that obtained randomly (empirical *p <* 0.01 using random sampling without replacement). This means that changes on those genes are less tolerated hence, more likely to cause disease [16].

We then queried the Clinical Variation (ClinVar) database [17] to assess the involvement of the 21 CDD genes carrying de novo variants in other human disorders. Six out the 21 genes are involved in human genetic diseases –mostly developmental-(*PXDN, RTEL1, ANKRD11, MAP2K1, CYLD* and *ACAN)* where epithelial, connective or bone morphologies being a common denominator (Additional file 1: Table S2). Protein truncating variants were detected for *PXDN, RTEL1, ANKRD11* and *PPP2R2B*. Importantly a damaging inherited mutation was also detected in the de novo mutated *TXLNB* gene.

CDD patients with mutations in genes known to be involved in other congenital human disorders were clinically re-assessed. Except for patient CC4C (*MAP2K1* damaging variant), in whom some developmental delay features had already been observed, none of the CDD patients showed signs of the disorders attributed to the genes in which they had damaging de novo variants. Importantly, the finding of a *MAP2K1* (Noonan's syndrome [18, 19]) damaging variant (c. 308 T > G, p.I103S) in patient CC4D helped confirm the diagnosis of the patient with Noonan's syndrome. CDD and keratosis pilaris (skin disorder) have also been reported in several Noonan’s syndrome patients [20–22].

Five genes with de novo damaging alleles *PIK3CA, TLN1 CYLD,* and *MAP2K1,* are linked with cholangio- and hepatocellular carcinomas in addition to congenital syndromes and conditions related to tissue overgrowth [23–26]. It is worth remarking that people affected with Brooke-Spiegler syndrome or familial cylindromatosis are born with one damaging allele in *CYLD* but a second damaging variant (somatic) needs to occur in the epithelial tissues for the disease to manifest, a process reminiscent of cancer development.

#### Genes carrying de novo variants enriched in significantly mutated regions (SMRs) in cancers

A recent study has summarized significantly mutated regions (SMRs) in 21 types of cancer [10]. In total, these regions comprise 610 genes thought to carry the “driver” mutations in different types of cancers. We found that de novo variants in SMR genes were in excess in our dataset (enrichment test *p <* 0.0249). This enrichment was due to the de novo variants in *PIK3CA*, *C6* and *PPP2R2B. PIK3CA* was highlighted by its very high density of mutations affecting 9 types of cancers, suggesting its key role in cancer development.

#### Inherited variants: homozygous, compound homozygous and di-genic models

We then assessed the patients for inherited damaging variants acting in a recessive manner. To identify compound heterozygotes (CH) we performed a gene burden test whereby those CH with *p* < 0.01 present in more than one patient (recurrently mutated) were considered.

CH recurrent events with at least one damaging allele were identified for 4 genes (*DCHS1, C5orf42*, *TXLNB* and, *PRRC2A)* and distributed among 7 families (Additional file 1: Table S2). As above, we queried the ClinVar database. *DCHS1* was found to have 4 different alleles forming two different CH in two different families (CC205 and VC84). In spite of *DCHS1* being linked to Van Maldergem Wetzburger Verloes syndrome, an autosomal recessive disorder characterized by intellectual disability, typical craniofacial features, auditory malformations resulting in hearing loss, and skeletal and limb malformations, neither patient had any sign of that syndrome. *DCHS1* is a key member of the planar cell polarity pathway which is involved in the regulation of the **cytoskeleton** that shapes the cells during mammalian development. Similarly, *C5orf42* was also found to have 4 different alleles forming two different CH in two different families (CC35 and CC9). This gene is associated with several developmental delay disorders including some forms of the autosomal recessive Joubert syndrome, global developmental delay and with the autosomal recessive orofaciodigital syndrome VI (polydactyly, cleft lip/palate or lingual lump, and psychomotor retardation). Neither *TXLNB* nor *PRRC2A* is linked to any human disease. *TXLNB* was also de novo mutated in patient VC10 and a homozygous *PRRC2A* mutation was identified in patient CC98.

Similar to the filtering strategy adopted for CH events, both recurrence and burden were considered for PPI pairs. After filtering, a total of 5 PPI combinations (*KRT18 + BYSL, THBS1 + COL7A1, TP53 + SETD8, EPS15 + DNM1,* and *POU2F2 + PGR*) distributed among 10 families were detected. All these genes are known to play pivotal roles in development.

*KRT18* encodes a protein for making structural keratin in hepatocytes. *KRT18* mutations cause infantile liver cirrhosis. *Krt18* mutant mice develop chronic hepatitis and hepatocyte fragility in association with disruption of hepatocyte keratin filaments mouse models [27]. *BYSL,* together with keratin 18 are thought to be involved in teratocarcinoma.

*THBS1* encodes a protein that mediates cell-to-cell and cell-to-matrix interactions and interacts with several proteins including the structural proteins encoded by *COL7A1. THBS1* has been shown to play roles in platelet aggregation, angiogenesis, and tumorigenesis and linked to auto immune disorders (Sjogren Syndrome). Mutant mice show abnormal cystic duct morphology, dilated gall bladder, abnormal pancreas morphology and pancreas inflammation [28]. *COL7A1* mutations are linked to two skin disorders, epidermolysis bullosa and keratosis palmoplantaris.

*TP53* germline mutations are linked to Li-Fraumeni syndrome, a rare autosomal dominant disorder that increases the risk of developing several types of cancer, particularly in children and young adults. *SETD8* regulates *TP53* and is involved in cell cycle progression and developmental processes.

No human disorders have been ascribed to the epidermal growth factor receptor *EPS15*. Dynamin (*DNM1*) encodes a microtubule-binding protein and been associated with early infantile epileptic encephalopathy. Little is known about the remaining complexes.

The identification of a gene with different damaging variants in different individuals (recurrently mutated gene) also evidences pathogenicity [29]. In total, we identified 13 genes recurrently mutated, forming either PPI or CH and as well as de novo or in homozygosis. Importantly, the alleles of recurrently mutated genes are not necessarily the same (gene mutated at different sites). Eight of the 13 recurrently mutated genes are present in different allelic forms (marked by * in Additional file 1: Table S2).

As to the phenotype caused by these mutated genes in mice, there are 9 genes (4 with de novo *ACAN, CYLD, PIK3CA, PPP1R15B*, and 5 with inherited variants *DCHS1, KRT18, PGR, THBS1,* and *TP53*) whose mouse mutants display abnormal liver or pancreas (including abnormal cystic duct and pancreas inflammation among others; Additional file 1: Table S2).

#### Gene-based and gene-set-based association test

Gene-based association tests were performed on the genes carrying rare and damaging variants (refer to Additional file 1: Figure S2). The *p*-values appear to be well estimated (as indicated by a qq-plot; Additional file 1: Figure S1), the good agreement with null distribution indicates no major biases due population stratification or technical issues. No signal reached the significance threshold of <10^−6^ for 20,000 genes tested although *TRIM28* achieved a significance level of 3.9 × 10^−5^, suggestive of enrichment in rare damaging variants (Additional file 1: Table S3).

To boost power for association analysis, we attempted to account for biological or functional relatedness by resorting to PPI (STRINGDB) and pathway (MsigDB) databases to group rare variants for association test. Pathway enrichment testing yielded no significant results.

Interestingly, PPI analyses revealed that the PPI pair *TRIM28* and *ZNF382* were overrepresented in the patient group when compared to controls (*p* < =3.60x10^−6^, Bonferroni corrected *p* < =0.042; Table 2). There are 3 individuals carrying rare damaging variants on *TRIM28* and 2 individuals with rare damaging variants on *ZNF382* (Additional file 1: Table S1). This indicates the role of *TRIM28* as a putative significant player in CDD. *TRIM28* is known to regulate endoderm differentiation including liver and pancreas structures during early embryogenesis [30]. *Trim28* knockout mice are embryonic lethal and show abnormal development of the digestive system and neural tube [31]. Until recently, studies revealed the role of *TRIM28* on genomic imprinting during embryo development [32], it has provided sights on its role in the developmental process.

**Table 2:** Association results for PPI gene-sets

PPI pairs	Markers tested	Empirical *p*-value
*TRIM28 + ZNF382*	13	3.60 × 10^−6^
*TRIM28 + KDM1A*	9	1.17 × 10^−5^
*TRIM28 + ZNF274*	20	2.93 × 10^−5^
*TRIM28 + HDAC1*	9	4.30 × 10^−5^
*TRIM28 + DNMT3A*	8	4.35 × 10^−5^
*TRIM28 + RNF4*	9	5.61 × 10^−5^
*TRIM28 + SERTAD1*	10	6.22 × 10^−5^
*TRIM28 + HTRA2*	10	6.23 × 10^−5^
*TRIM28 + TOPORS*	8	6.72 × 10^−5^
*IRF1 + TRIM28*	8	6.86 × 10^−5^
*TRIM28 + IRF7*	9	6.95 × 10^−5^
*TRIM28 + ZNF689*	10	8.62 × 10^−5^
*TRIM28 + VEZF1*	9	9.40 × 10^−5^
*TRIM28 + UBE2U*	8	9.76 × 10^−5^
*TRIM28 + MIS12*	12	1.05 × 10^−4^
*RUVBL2 + TRIM28*	10	1.26 × 10^−4^
*TRIM28 + TRIM33*	10	1.30 × 10^−4^
*TRIM28 + ZNF354A*	14	1.57 × 10^−4^
*TRIM28 + SOCS1*	9	1.83 × 10^−4^

*TRIM28* is also listed among 610 SMR genes in cancer.

#### Searching functional overlap among genes

**Fig. 2:**
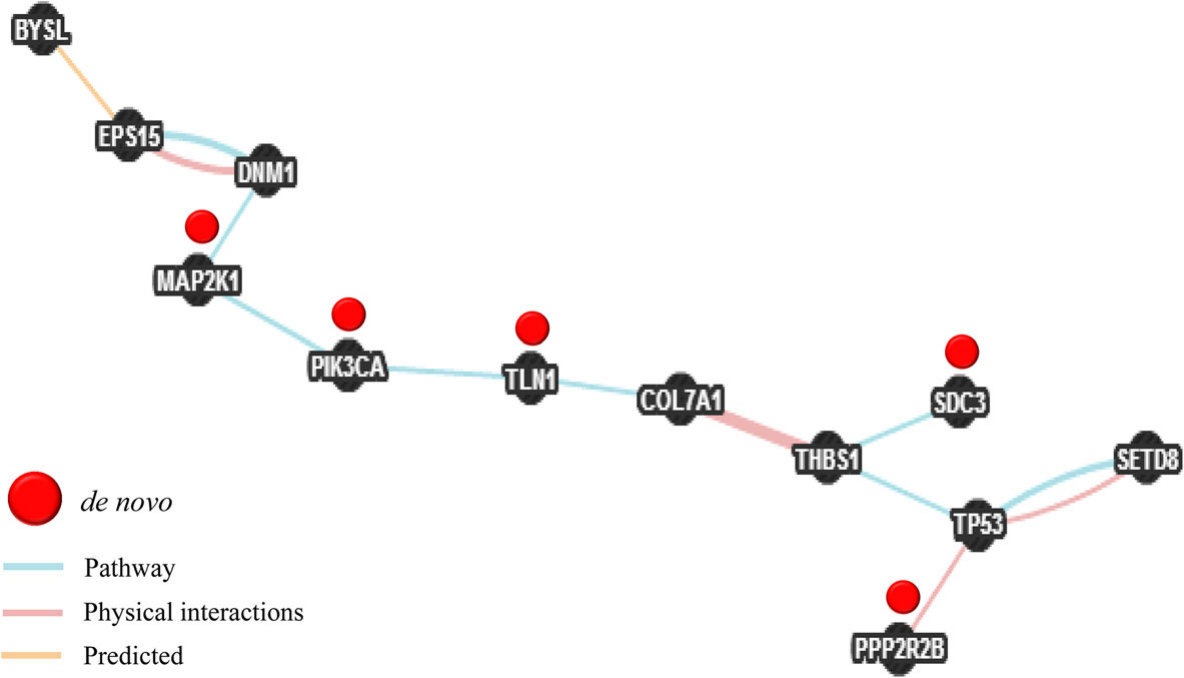
Biological network integration of genes by GeneMania. All genes with variants were submitted for functional network analyses. Twelve interlinked genes were identified. Five with de novo variants (*MAP2K1, PIK3CA, TLN1, PPP2R2B, SDC3*) and 7 recurrently mutated *(BYSL, EPS15, DNM1, COL7A1, THBS1, TP53, SETD8*)

In order to fully understand how this myriad of mutated genes identified in our patients could contribute to the CDD phenotype and to consider whether our findings fit into any pathological process, we performed gene/pathway-set enrichment analyses and a careful examination of the genetic profile or mutational load of each patient. No enriched geneset was found using DAVID [33]. However, GeneMANIA [34] identified 12 genes being part of the same network as indicated by their pathway and physical interactions (Fig. 2). Among these, 5 had de novo variants and 7 were recurrently mutated (forming CH or PPIs) at different sites.

Additional file 1: Table S2 presents the genetic profile of each of the 23 CDD patients with damaging alleles, the involvement of the mutated gene in other human disorders and the phenotype displayed by mutant mice. Several patients had more than one de novo variants and/or more than one damaging allele advocating the idea of CDD being an oligogenic and genetically heterogeneous disorder and as such, the phenotype will be variable and result from gene interactions. Hence the challenge in disentangling such disorders.

Seven patients had no damaging variants (in bold in Additional file 1: Table S1). Among these, patients CC221C and CC232C had concomitant developmental disorders. Not finding damaging alleles indicates i) we missed them; ii) those patients may have small chromosomal anomalies or copy number variations (CNV) or, (iii) non-genetic disease cause. Patient CC7, had a de novo frameshift in *PPP2R2B,* a gene linked to spinocerebral ataxia, and inherited damaging alleles in the PPI pair *THBS1* + *COL7A1.* Whether *PPP2R2B* will eventually confer the spinocerebral ataxia phenotype (late onset) to that patient and the CDD is caused by the variants in PPI is not known. The patient is being monitored.

## Discussion

The two current theories for the aetiology of CDD, “obstructing segment” hypothesis and the “pancreatic reflux” hypothesis are not mutually exclusive. Both imply that CDD results from a failure in the hepatobiliary-pancreatic development. Given the multitude of molecular and cellular events that take place during the development of the hepatobiliary system, it is conceivable that DNA alterations in genes governing such processes could underlie CDD. As the number of interacting molecules during development is large, CDD may result from the accumulation of mutations in several genes. Variants in different genes of related pathways may lead to the same disorder, the same gene may have different severe variants in unrelated patients or, a given variant may lead to different clinical manifestations in different individuals. Hence, the marked genetic heterogeneity that characterizes human diseases [35, 36].

The genetic profile of these CDD patients indicates the disorder is not only genetically heterogeneous but also oligogenic, comprising di-genic models, and is consistent with the sporadic presentation of the disease and the scarcity of familial cases. Although some of the genes with de novo pathogenic mutations appear to be functionally or biologically linked with developmental processes of the hepatobiliary tract, it would appear that CDD obeys a “two or multi-hit hypotheses” where the effect of de novo variants may be “enhanced” by the presence of damaging alleles of genes other than those with the de novo variant. These damaging alleles may form compound heterozygotes and/or be PPI-related. As CDD is more common in Asians, the frequencies of alleles conferring risk to the disorder in that population are likely to be higher than in other ethnicities. The presence of an underlying genetic risk factor seems to be corroborated by our gene-enrichment test whereby we evaluated the contribution of rare damaging variants by a gene-based case–control association analysis [36]. These rare variants could modify the manifestation of the disease by enhancing or reducing the effect of other co-existing alleles and provide the extra stimulus that drives those susceptible individuals to disease. Thus, it would appear that carriers of *TRIM28* or *ZNF382* rare damaging variants might have a higher risk of developing CDD. Replication of this finding in independent Chinese patients is needed. Similar studies should be conducted in non-Asian CDD patients to assess whether the *TRIM28* association is population specific.

Interestingly, multiple CDD genes coincided with SMR cancer genes as suggested by the enrichment of SMR genes with de novo variants and the existence of our top associated gene-pair (*TRIM28-ZNF382*) among the SMR list in our CDD samples. Our data is in line with the established link between cholangiocarcinoma and CDD. Importantly, our literature review showed that CDD is not only linked to cholangiocarcinoma but also to other types of malignancies, including adenocarcinomas, primary squamous carcinoma of the liver, benign squamous metaplasia and adenosquamous carcinoma [37–39].

We then investigated if of those genes with damaging variants (either de novo or inherited) were biologically and/or functionally related and the phenotypes that these genes originate, when mutated, in human and mice. Twelve genes with damaging alleles were found to physically or functionally interact (Fig. 2). As for the phenotypic effect in human or mice, 6 genes carrying de novo have been associated with human developmental disorders that involve overgrowth of the epithelial and/or connective tissues, bone morphologies (Additional file 1: Table S2). Those genes not reported implicated in human disorders (*N* = 15) are all involved in cell-to-cell and cell-to-matrix interactions and in maintaining the structural integrity of the epithelial cells.

Except for one patient with Noonan’s syndrome, none of the patients displayed any phenotype other than CDD at the time of diagnosis. We can only speculate that, allegedly, unrelated disorders may also be genetically connected as indicated by the human disease network map (or diseasome) [40, 41].

The excess of damaging alleles in genes mainly involved in soft tissues disorders and conditions related to overgrowth of tissues (benign or cancerogenous) is in line with i) the clinical observation regarding the higher incidence of cholangiocarcinomas among CDD patients; ii) the notion that CDD is caused by tissue overgrowth which would fit with the “obstructing segment” and the “pancreatic reflux” hypotheses on the CDD aetiology. Moreover, the cholangiocyte function is likely to be affected and so, the bile composition which in turn may cause further damage to the epithelium. These hypotheses are not mutually exclusive. Either stenosis or pancreatico-biliary junction defect can underlie CDD and which of them triggers the disease would depend on the genetic background of the patient.

Despite the array of databases and prediction tools available, very little is known about the intricate ways in which genes interact, the development of the hepato-biliary structures, or the function of many genes. For example, one de novo and two inherited damaging alleles in an evolutionarily constrained gene, *TXLNB,* were identified in two patients. Yet, very little is known about this gene. However, finding genes with rare damaging variants at different sites in different individuals (gene recurrently mutated) can be used as a “pathogenicity criteria” to ascribe the involvement of a gene in a disorder [42]. For rare, genetically heterogeneous diseases such as CDD, the involvement of a mutated gene in the disease may only be consolidated by the identification of new or recurrent mutations of the same gene in independent patients.

Ours is the first genetic study ever conducted on CDD patients. The study is not without limitations, small sample size being the most obvious. Yet, genes functionally linked and recurrently mutated have been identified providing a glimpse into the genetic factors that may be underlying CDD. For such a rare and genetically heterogeneous disorder, replication of our findings implies unattainable sample size. Also, our data indicates that the phenotype results from interactions between more of one mutated gene (digenic/oligogenic) which hampers the possibility of a clean functional study.

## Conclusions

Our data supports a strong genetic basis for CDD and show that CDD is not only genetically heterogeneous but also non-monogenic, requiring mutations in more than one genes for the disease to develop. The data is consistent with the rarity and sporadic presentation of CDD.

## Additional file

Additional file 1: Supplementary information. (DOC 653 kb)

## Abbreviations

CBD: Common biliary duct
CDD: Congenital dilatation of the bile-duct
CH: Compound heterozygous
DDD: Degenerative disc disease
MAF: Minor allele frequency
MRCP: Magnetic resonance cholangiopancreatography
PKD: Polycystic kidney disease
PPI: Protein-protein interaction
RVTEST: Rare variant test software for next generation sequencing data
SKAT: Sequence kernel association test
SMR: Significantly mutated regions (in cancers)
SNV: Single nucleotide variant
WES: Whole exome sequencing
