# Supplementary material for: Genetic study of congenital bile-duct dilatation identifies de novo and inherited variants in functionally related genes
Source: BMC Med Genomics. 2016 Dec 12;9:75. doi: 10.1186/s12920-016-0236-z (PMC5154011; doi:10.1186/s12920-016-0236-z)
Supplement: Additional file 1: — Supplementary information. (DOC 653 kb) [file 12920_2016_236_MOESM1_ESM.doc]

**TITLE:**

Genetic study of congenital bile-duct dilatation identifies de novo and inherited variants in functionally related genes

**AUTHORS:**

John KL WONG^1^, Desmond CAMPBELL^1^, Ngoc Diem NGO^2^, Fanny YEUNG^3^, Guo CHENG^3^, Clara SM TANG^3^, Patrick HY CHUNG^3^, Ngoc Son TRAN^2^, Man-ting SO^3^, Stacey S CHERNY^1,4^, Pak C SHAM^1,4,5^, Paul K TAM^3,5^, Maria-Mercè GARCIA-BARCELO^3,5,*^.

# AFFILIATIONS:

^1^Department of Psychiatry, Li Ka Shing Faculty of Medicine, The University of Hong Kong, Hong Kong SAR China; ^2^National Hospital of Pediatrics, Hanoi, Viet Nam; ^3^Department of Surgery, Li Ka Shing Faculty of Medicine, The University of Hong Kong, Hong Kong SAR, China; ^4^Center for Genomic Sciences, Li Ka Shing Faculty of Medicine, The University of Hong Kong, Hong Kong SAR, China; ^5^Centre for Reproduction, Development, and Growth, Li Ka Shing Faculty of Medicine, The University of Hong Kong, Hong Kong SAR, China^5^

Table of Contents

[SUPPLEMENTARY DATA 3](#__RefHeading___Toc433120818)

[Material and Methods (refer to supplementary Figure 2 for flowchart) 3](#__RefHeading___Toc433120819)

[Whole Exome Sequencing (WES) 3](#__RefHeading___Toc433120820)

[Quality control (QC) and variant calling 3](#__RefHeading___Toc433120821)

[Bioinformatics analyses 5](#__RefHeading___Toc433120822)

[SUPPLEMENTARY FIGURES 8](#__RefHeading___Toc433120823)

[Supplementary Figure S1: Quantile-quantile plot from gene based test 8](#__RefHeading___Toc433120824)

[Supplementary Figure S2: The flowchart of the exome sequencing analysis pipeline. 9](#__RefHeading___Toc433120825)

[SUPPLEMENTARY TABLES 10](#__RefHeading___Toc433120826)

[Supplementary Table S1: Clinical information of CDD patients included in the study 10](#__RefHeading___Toc433120827)

[Supplementary Table S2: List of variants under different hypothesis: *de novo*, compound heterozygous and PPI. 12](#__RefHeading___Toc433120828)

[Supplementary Table S3: Gene based SKAT test results 16](#__RefHeading___Toc433120829)

[REFERENCES 17](#__RefHeading___Toc433120830)

# SUPPLEMENTARY DATA

## Material and Methods (refer to supplementary Figure 2 for flowchart)

### Whole Exome Sequencing (WES)

Exome sequencing was performed at the Centre for Genomic Sciences (CGS) of the Li Ka Shing Faculty of Medicine of the University of Hong Kong on blood DNA. Illumina’s TruSeq® DNA Sample Prep v.2 and TruSeq® Exome Enrichment Kits (Illumina, San Diego, CA, USA) were used for sample preparation and capture, and enrichment of targeted sequences, respectively. The captured DNA was sequenced as paired-end 100 base reads (PE100) on an Illumina HiSeq 2000, aiming to achieve 121 reads per base (121X) in average.

### Quality control (QC) and variant calling

The quality assessment of sequencing reads starts with the raw reads. The base quality, duplication levels, GC bias and primer sequences of the raw sequencing reads were evaluated using FastQC. All samples passed the sequencing quality thresholds.

Afterwards, sequencing reads were aligned to the human genome reference hg19 by the Burrows-Wheeler Aligner (BWA v 0.7.8) to produce the sequence alignment file. For the detection of contaminated samples, we used the verifyBAMID software. This program takes a sequence alignment file and the 1000 genome reference sites file as input to evaluate the heterogeneity of each given site of our dataset. Individuals reported with >3% contamination should be excluded in further analysis. There is one indexed patient failed the test (level of contamination ~11%) and was excluded.

Calling and filtering of single nucleotide variants (SNVs) and indels (small insertions/deletions) were done by the Genome Analysis Toolkit (GATK 3.3-0) haplotype-caller and Variant Quality Score Recalibration (VQSR) module respectively. VQSR compares known sites in variant databases (1000 Genome and dbsnp137) with novel variants on a number of sequencing parameters in our dataset. This aims to investigate relationships between sensitivity and specificity of variants calling. There are 7 sensitivity trenches defined between 90% and 100% in our dataset. After plotting the relationship between sensitivity and specificity, an optimal VQSR sensitivity tranche was determined to be <=99.5 and therefore variants in trenches with >99.5 sensitivity were excluded. Then we looked at the variants of each individual sequenced. Hard filtering on parameters such as genotype calls were performed by KGGSeq on each individual variant. Variants with depth of coverage <=4, genotyping quality <20 were set as missing.

Principal component analysis (PCA), implemented in PLINK 1.9, was used for the detection of population stratification. Sample relationship checks were performed in pairwise fashion by PLINK, there is one trio subjected to non-maternity issue and therefore excluded from all family based analysis.

For the purpose of association testes, we evaluated the genotypes at “genomic site level” and removed low confidence variations. Additional criteria for good genotyping quality include: low missingness and conforming Hardy-Weinberg equilibrium (HWE). To achieve these, PLINK was used to filter out sites with >=10% missing rate and sites failed a HWE test (*p*<0.0001). Then, to enhance the calling accuracy and to facilitate the filtering process, 700 samples from local Chinese individuals participating in the degenerative disc disease (DDD) exome sequencing projects were added to the CDD calling set. These samples had also been sequenced at the Centre for Genomic Sciences (CGS) and processed by a similar exome sequencing pipeline. Principal component analysis (PCA) indicated no observable bias between CDD samples sequenced and these DDD control samples. The resulting variants were subjected to further selection as described in the next section.

Sanger sequencing was used for validation of selected non-synonymous *de novo* and inherited variants.

### Bioinformatics analyses

We used KGGSeq for annotation and pathogenicity assessment of single nucleotide variants (SNVs) and small insertions or deletions (INDELs). KGGSeq makes use of valuable biologic resources to provide a comprehensive and efficient framework to filter and prioritize genetic variants from WES data. KGGSeq integrates 4 prediction programs (Polyphen2, Sift, MutationTaster and Likelihood ratio) which are weighted by logistic regression. To integrate the results under different disease models, we used “gene-level” annotation which assesses the phenotypic effect of the “mutated” gene (irrespectively of the variant) in mice and in humans by resorting to databases such as Mouse Genome Informatics (MGI), OMIM, ClinVar, Uni-prot and others.

The annotated list of variants was filtered firstly by the type of variants whereby synonymous variants were excluded. Also, filtering for MAF <=1% using 1000GP, ESP6500 and dbsnp137 were applied to restrict our analysis to rare variants. Those SNVs or Indels in genes evolutionary constrained were prioritized as genes with positive constraint scores are subjected to higher evolutionary pressure and therefore variants in those genes are more likely to be pathogenic (Supplementary Table S2).

To further assess if those selected evolutionary constrained genes with variants could be grouped by functional categories we used The Database for Annotation, Visualization and Integrated Discovery (DAVID). DAVID produces False Discovery Rate (FDR) corrected *p*-values for each gene set. GeneMania was used to create and visualize gene networks by evidence in pathways and protein-protein interactions (predicted and experimental).

*Constraint scores*

The selective constraint scores used in this manuscript were previously described and were provided to us by the author (personal communication with Dr. Neale). In brief, constraint scores were calculated by a mutation rate table that contain the probability of every triplets mutating to every other combination of triplets by only changing the third nucleotide. The calculation was based on intergenic SNPs from the 1000 Genomes project in account for the gene length to determine that gene’s probability of mutation. These probabilities of mutation were also corrected for regional divergence and depth of coverage for each base by exome sequencing study included in the calculation. Since there is a high correlation (Pearson’s r = 0.94) between the probability of a synonymous mutation with the number of rare (MAF < 0.01%) synonymous variants in the same gene seen in the NHLBI’s Exome Sequencing Project, they have used a linear model to predict the number of rare missense variants expected per gene in the same dataset. The difference between observation and expectation was quantified as a signed Z score under chi-squared distribution. Thus, the calculated missense Z score can be used as the basis for determining selective constraint.

# SUPPLEMENTARY FIGURES

## Supplementary Figure S1: Quantile-quantile plot from gene based test


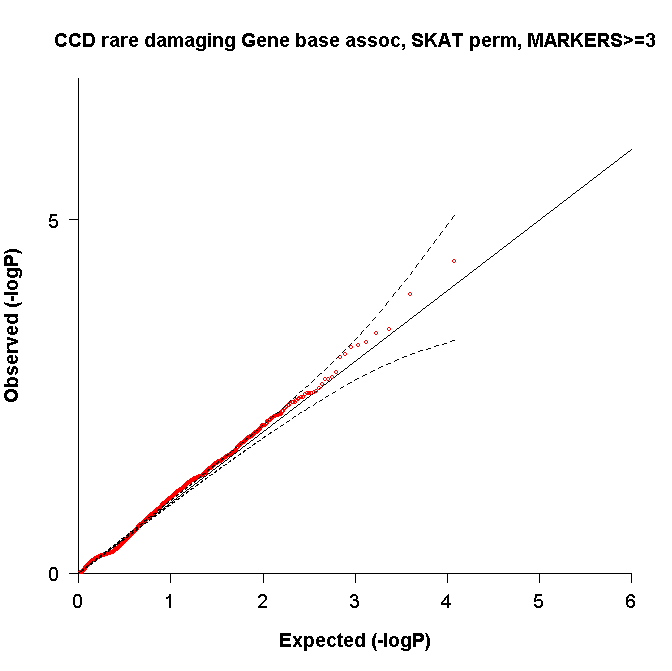
The quantile-quantile plot below was produced from the *p*-values of the gene-based association test using SKAT. Only genes with more than 2 markers were plotted. The *p*-values follow the null well, which shows no bias in the tested dataset.

## Supplementary Figure S2: The flowchart of the exome sequencing analysis pipeline.

DP: Coverage depth, GQ: Genotype quality


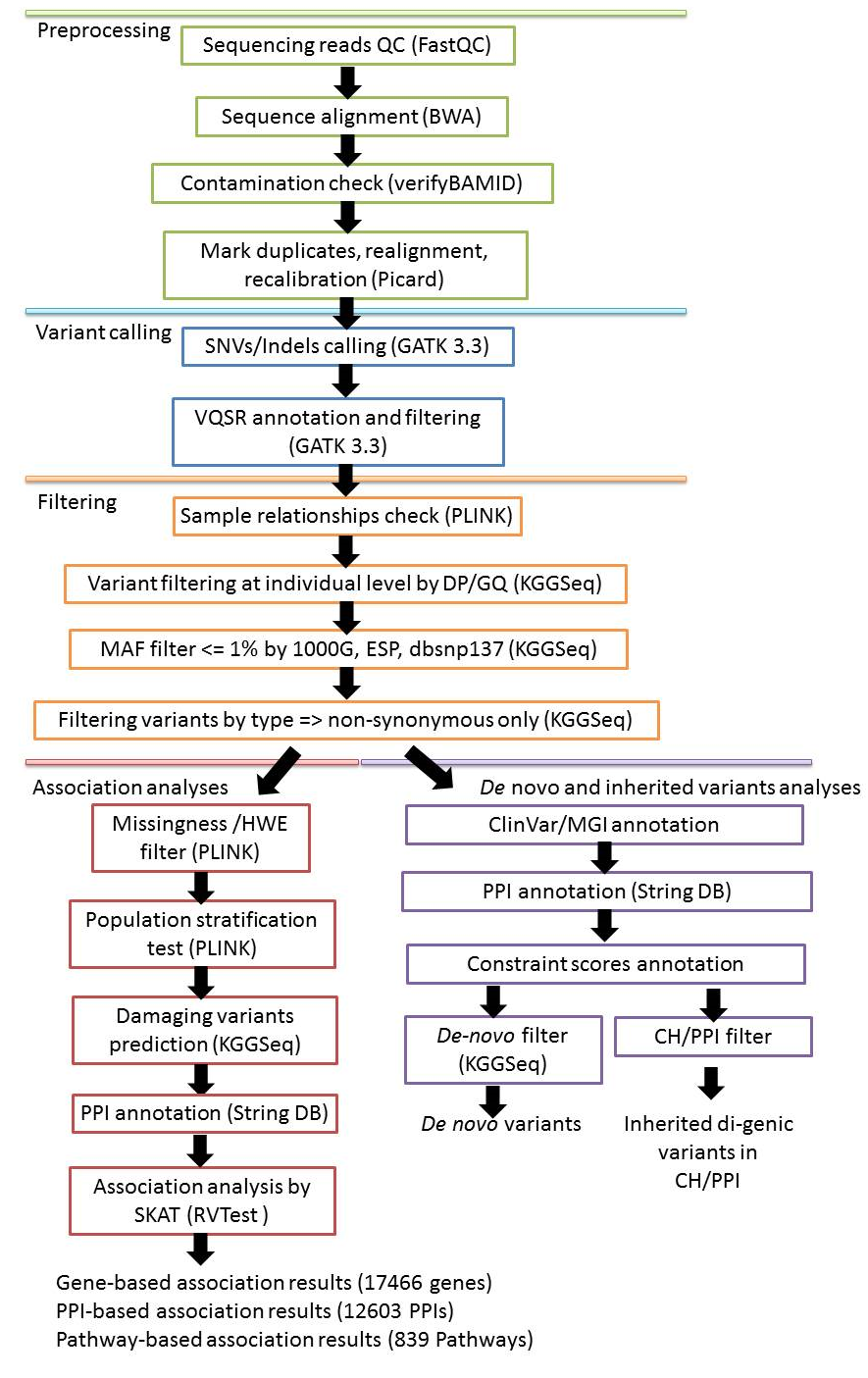


# SUPPLEMENTARY TABLES

| Supplementary Table S1: Clinical information of CDD patients included in the study | | | | | |
| --- | --- | --- | --- | --- | --- |
| **ID** | **Sex** | **Type of CCD** | **Diagnosis (AN/PN)** | **Other concomitant disorders** | **Anatomical features** |
| CC3 | F | I | PN | Nil | CBD fusiform dilatation; not involving bifurcation. Pancreatic duct (PD) not seen |
| CC4 | F | I | PN | Noonan’s syndrome | Dilated cystic duct; long segment of non-dilated common channel ~8mm at lower end of CBD; proteinous plug in PD |
| CC7 | F | I | AN | Nil | CBD fusiform dilatation with proximal extension of ductal dilatation in common hepatic duct; no PD involvement |
| CC9 | F | I | PN | Nil | CBD saccular dilatation; extends proximally to confluence of intrahepatic ducts and proximal right and left intrahepatic ducts. Pancreatic duct displaced anteriorly and appears to join the CBD at distal end of the CC |
| CC10 | F | IV | PN | Nil | CC with dilatation of extrahepatic and intrahepatic bile ducts. Gallstone in gallbladder; PD not dilated; anatomy at junction of CBD and PD are not well delineated |
| CC14 | F | I | PN | Nil | CBD dilatation; PD at lower end of CC with long common channel |
| CC25 | F | I | PN | Nil | CBD saccular dilatation with extension into proximal right and left intrahepatic ducts; Ductal stone in distal CBD; PD at lower end of CBD with common channel 8mm length |
| CC29 | F | I | AN | Nil | CBD dilatation; no intrahepatic duct involvement |
| CC30* | F | I | AN | Nil | CBD dilatation with proximal extension to confluence; intrahepatic ducts undilated |
| CC35 | F | I | PN | Nil | No information |
| CC36 | F | I | PN | Nil | CBD saccular dilatation with proximal extension to confluence and right/left intrahepatic ducts; Gallbladder sludge; PD not dilated |
| CC37* | F | I | PN | Nil | CBD fusiform dilatation; no proximal extension; PD cannot be seen clarly |
| **CC39** | **F** | **I** | **AN** | **Nil** | **CBD dilatation; no intrahepatic duct involvement; PD not dilated** |
| CC55 | F | I | PN | Nil | CBD dilatation with proximal extension to confluence; intrahepatic ducts undilated; PD not dilated |
| CC81* | M | I | PN | Nil | CBD fusiform dilatation with no proximal extension; PD not dilated with long common channel 11mm |
| **CC97** | **F** | **I** | **PN** | **Nil** | **CBD fusiform dilatation with no proximal extension; PD not well seen** |
| CC98 | F | I | AN | Nil | CBD dilatation with no proximal extension; PD not well seen |
| CC205 | F | I | AN | Nil | CBD saccular dilatation, no proximal extension; PD not dilated |
| **CC221** | **F** | **I** | **AN** | **Patent ductus arteriosus; pulmonary stenosis.** | **CBD dilatation with no proximal extension; PD not well seen** |
| CC226 | F | I | AN | Nil | CBD dilatation with no proximal extension; PD not well seen |
| **CC229*** | **F** | **I** | **PN** | **Nil** | **CBD dilatation with extension into left intrahepatic ducts** |
| CC231 | F | I | AN | Nil | CBD fusiform dilatation, with no proximal extension, PD not dilated |
| **CC232** | **F** | **I** | **PN** | **Dyspraxia** | **CBD fusiform dilatation with no proximal extension; PD not well seen** |
| **CC233** | **F** | **I** | **PN** | **Nil** | **NA** |
| CC234 | F | I | PN | Nil | NA |
| VC10C | F | IV | PN | Nil | NA |
| VC18C | F | IV | PN | Nil | NA |
| VC49C* | F | I | PN | Nil | NA |
| VC61C | F | IV | PN | Nil | NA |
| VC63C | F | IV | PN | Nil | NA |
| **VC65C** | **F** | **I** | **PN** | **Nil** | **NA** |
| VC84C | F | I | PN | Nil | NA |
| **VC88C** | **F** | **I** | **PN** | **Nil** | **NA** |
| CC: sample recruited in Hong Kong; VC: sample recruited in Hanoi, Viet Nam; CBD: common bile duct, PD: pancreatic duct; AN: antenatal, PN: postnatal; NA: information not available. In bold: patients with no damaging variants; *: patients with *TRIM28* or *ZNF382* damaging variants. | | | | | |

| Supplementary Table S2: List of variants under different hypothesis: *de novo*, compound heterozygous and PPI. |
| --- |
| | **Family** | **Type** | **Genes** | **MAF** | **Variant type** | **Protein change** | **Constraint score** | **Human Disease**  **(From ClinVar)** | **Mouse phenotype**  **(hepatobiliary/pancreas/cysts)** | | | --- | --- | --- | --- | --- | --- | --- | --- | --- | --- | | **VC84** | **CH** | *DCHS1** | N/N | M/M | p.A2955S/ p.P2750S | 1.92 | Van Maldergem Wetzburger Verloes syndrome (AR)**^θ^** | abnormal intestine morphology, kidney cysts | | | **PPI** | *TP53+SETD8* | 0.0018/RNF | M/M | p.V31I/ p.L332P | 1.52/2.19 | Carcinoma of pancreas, Dyskeratosis congenita (AR)**+^** / NA | increased liver and pancreatic carcinoma incidence / NA | | | **VC63** | **PPI** | *EPS15*+DNM1** | 0.0018/0.03 | M/M | p.D705N/ p.L16M | 0.88/4.2 | NA / Epileptic encephalopathy | NA / NA | | | *de novo* | *ZNF330* | N | M | p.*F54L* | 0.5 | NA | NA | | | **VC61** | *de novo* | *CYLD* | N | M | p.W487L | 3.16 | Familial multiple trichoepitheliomata or Spiegler-Brooke syndrome (AR)**+^**, Cylindromatosis | abnormal intestinal epithelium morphology | | | *de novo* | *KCNH3* | N | M | p.V195A | 3.61 | NA | NA | | | *de novo* | *SDC3* | N | M | p.R302W | 0.41 | NA | NA | | | **VC49** | **CH** | *PRRC2A* | 0.0047/0.0046 | M/M | p.V961A/ p.L1980I | 3.65 | NA | NA | | | **VC10** | *de novo* | *PPP1R15B* | N | M | p.P139H | 0.99 | NA | abnormal liver morphology | | | *de novo* | *RTEL1* | N | S | p.G973* | -1.14 | Dyskeratosis congenital (AR/AD)**+^**, Pulmonary fibrosis (AR) | NA | | | *de novo* | *TXLNB* | 0.0002 | M | p.R211Q | -0.02 | NA | NA | | | **CC98** | **Homozygous** | *PRRC2A* | 0.0046 | M | p.L1980I | 3.65 | NA | NA | | | **CC9** | **CH** | *C5orf42** | N/0.0014 | M/M | p.S2287N/ p.M539T | -0.16 | Joubert syndrome (AR) **^θ^**,Global developmental delay,Orofaciodigital syndrome VI (AR) **^θ^** | kidney cysts | | | **CC81** | *de novo* | *C6* | N | M | p.W571C | -0.42 | Complement component 6 deficiency | NA | | | *de novo* | *HEATR6* | N | M | p.T948I | -0.12 | NA | NA | | | *de novo* | *PIK3CA* | N | M | p.I191M | 4.1 | Keratosis seborrheic (AD)**+^**, cancer | increased pancreatic beta cell number | | | **PPI** | *POU2F2*+PGR* | N/N | M/M | p.Y87H/ p.L459F | N/A | NA / NA | NA / abnormal pancreatic beta cell physiology,  increased pancreatic beta cell proliferation | | | **CC7** | *de novo* | *PPP2R2B* | N | F | p.G386fs | 2.28 | Spinocerebellar ataxia (AD) | NA | | | **PPI** | *THBS1*+COL7A1* | N/0.0014 | M/M | p.V21L/Q152R | 1.94/0.4 | Sjogren Syndrome**^+^** / epidermolysis bullosa (AR)**+^**; keratosis palmoplantaris (AD)**+^**; | abnormal cystic duct, pancreas morphology, pancreas inflammation / NA | | | **CC55** | *de novo* | *ANKRD11* | N | M | p.K1464fs | 2.87 | KBG syndrome (AD) **^θ+^** |  | | | *de novo* | *TLN1* | N | M | p.R2398W | 4.52 | NA | NA | | | **CC4** | *de novo* | *MAP2K1* | N | M | p.I103S | 2.76 | Noonan's syndrome (AD, AR) **^θ+^**, intrahepatic cholangiocarcinoma | NA | | | **CC37** | **PPI** | *POU2F2*+PGR* | N/N | M/M | p.E224K/ p.L459F | N/A | NA / NA | NA / abnormal pancreatic beta cell physiology,  increased pancreatic beta cell proliferation | | | **CC36** | **PPI** | *KRT18+BYSL** | N/N | M/M | p.R131C/ p.V123D | 2.31/0.95 | Cirrhosis, Teratocarcinoma/ Teratocarcinoma | enlarged liver / NA | | | **CC35** | **CH** | *C5orf42** | 0.0046/N | M/M | p.A2916T/ p.A579V | -0.16 | Joubert syndrome (AR) **^θ^**,Global developmental delay,Orofaciodigital syndrome VI (AR) **^θ^** | kidney cysts | | | *de novo* | *MSANTD3-TMEFF1* | N | M | p.H231R | 1.25 | NA | NA | | | *de novo* | *PXDN* | N | F | p.P225fs | 3.35 | Sclerocornea (AD) | NA | | | **PPI** | *TP53+SETD8* | 0.0018/RNF | M/M | p.V31I/ p.L332P | 1.52/2.19 | Carcinoma of pancreas, Dyskeratosis congenita (AR)**+^** / NA | increased liver and pancreatic carcinoma incidence / NA | | | **CC30** | **PPI** | *THBS1*+COL7A1* | N/0.0014 | M/M | p.P1795L/ p.Q152R | 1.94/0.4 | Sjogren Syndrome**^+^** / epidermolysis bullosa (AR)**+^**; keratosis palmoplantaris (AD)**+^**; | abnormal cystic duct, pancreas morphology, pancreas inflammation / NA | | | **CC3** | *de novo* | *TENM4* | N | M | p.R2238W | N/A | NA | NA | | | **CC29** | **PPI** | *KRT18+BYSL** | N/N | M/M | p.R131C/ p.A92P | 2.31/0.95 | Cirrhosis, Teratocarcinoma/ Teratocarcinoma | enlarged liver / NA | | | **CC25** | **CH** | *TXLNB** | N/0.000232558 | M/M | p.K229R/ p.V126I | -0.02 | NA | NA | | | **CC234** | *de novo* | *KRT80* | N | M | p.S249C | 0.2 | NA | NA | | | **CC231** | **PPI** | *EPS15*+DNM1** | 0.0018/0.03 | M/M | p.L16M/ p.N845S | 0.88/4.2 | NA / Epileptic encephalopathy | NA / NA | | | **CC226** | *de novo* | *ACAN* | N | M | p.C2282R | 0.78 | Spondyloepimetaphyseal dysplasia (AR) **^θ+^**, Osteochondritis dissecans**^θ+^** | abnormal liver morphology, enlarged liver | | | **CC205** | **CH** | *DCHS1** | N/N | M/M | p.S2116F/ p.M1771I | 1.92 | Van Maldergem Wetzburger Verloes syndrome (AR)**^θ^** | abnormal intestine morphology, kidney cysts | | | *de novo* | *PTGER3* | N | M | p.A158V | 3.55 | NA | NA | | | **CC14** | *de novo* | *PQLC2* | N | M | p.V2F | 1.56 | NA | NA | | | **AR: Autosomal recessive, AD: Autosomal dominant, *indicate variants carrying different alleles in their recurrence, M: Missense, S: Stopgain, F: Frameshift, θ:Disease related to bone development, +:Connective tissue disease, ^:Disease affecting skin** | | | | | | | | |  | | **The complete table of recurrent mutations under different hypotheses (*de novo*, compound heterozygous and PPI). Each variant were annotated by its possible functional impact and related mouse/human phenotypes, only phenotypes related to CCD were listed.** | | | | | | | | |  | |

| Supplementary Table S3: Gene based SKAT test results |
| --- |
| | **Gene name** | **Tested markers** | ***p*-values** | | --- | --- | --- | | ***TRIM28*** | 7 | 3.90x10^-5^ | | ***M1AP*** | 6 | 1.13x10^-4^ | | ***MASTL*** | 10 | 3.50x10^-4^ | | ***PHACTR2*** | 9 | 4.00x10^-4^ | | ***COL25A1*** | 4 | 5.50x10^-4^ | | ***KRTAP5-8*** | 5 | 6.00x10^-4^ | | ***KLC2*** | 8 | 6.50x10^-4^ | | ***FUT11*** | 5 | 8.00x10^-4^ | | ***PIWIL4*** | 10 | 9.00x10^-4^ | | ***OR13C8*** | 9 | 1.45x10^-3^ | | ***CHURC1*** | 4 | 1.70x10^-3^ | | ***CDC20*** | 6 | 1.80x10^-3^ | | ***ERGIC2*** | 3 | 1.80x10^-3^ | | ***FEM1C*** | 6 | 2.15x10^-3^ | | ***CAPRIN1*** | 5 | 2.45x10^-3^ | | ***MAEL*** | 6 | 2.70x10^-3^ | | ***TBCE*** | 7 | 2.80x10^-3^ | | **Gene-based association test was performed on genes with >=3 markers using SKAT. The top gene *TRIM28* achieved a significance level of 3.90x10^-5^, which is marginally significant.** | | | |

# REFERENCES

## 1. Andrews S: **FastQC: A quality control tool for high throughput sequence data**. *Reference Source* 2010.

## 2. Li H, Durbin R: **Fast and accurate long-read alignment with Burrows-Wheeler transform**. *Bioinformatics* 2010, **26**(5):589-595.

## 3. Jun G, Flickinger M, Hetrick KN, Romm JM, Doheny KF, Abecasis GR, Boehnke M, Kang HM: **Detecting and estimating contamination of human DNA samples in sequencing and array-based genotype data**. *American journal of human genetics* 2012, **91**(5):839-848.

## 4. McKenna A, Hanna M, Banks E, Sivachenko A, Cibulskis K, Kernytsky A, Garimella K, Altshuler D, Gabriel S, Daly M *et al*: **The Genome Analysis Toolkit: A MapReduce framework for analyzing next-generation DNA sequencing data**. *Genome research* 2010, **20**(9):1297-1303.

## 5. Li MX, Gui HS, Kwan JS, Bao SY, Sham PC: **A comprehensive framework for prioritizing variants in exome sequencing studies of Mendelian diseases**. *Nucleic acids research* 2012, **40**(7):e53.

## 6. Purcell S, Neale B, Todd-Brown K, Thomas L, Ferreira MA, Bender D, Maller J, Sklar P, de Bakker PI, Daly MJ *et al*: **PLINK: A Tool Set for Whole-Genome Association and Population-Based Linkage Analyses**. *AmJ HumGenet* 2007, **81**(3):559-575.

## 7. Li MX, Kwan JS, Bao SY, Yang W, Ho SL, Song YQ, Sham PC: **Predicting mendelian disease-causing non-synonymous single nucleotide variants in exome sequencing studies**. *PLoS Genet* 2013, **9**(1):e1003143.

## 8. Samocha KE, Robinson EB, Sanders SJ, Stevens C, Sabo A, McGrath LM, Kosmicki JA, Rehnstrom K, Mallick S, Kirby A *et al*: **A framework for the interpretation of de novo mutation in human disease**. *Nature genetics* 2014, **46**(9):944-950.
